# Supplementary material for: Radiation Retinopathy: Microangiopathy-Inflammation-Neurodegeneration
Source: Cells. 2025 Feb 18;14(4):298. doi: 10.3390/cells14040298 (PMC11853965; doi:10.3390/cells14040298)
Supplement: Supplementary file 1 [file cells-14-00298-s001.zip › Figure S1.pdf]

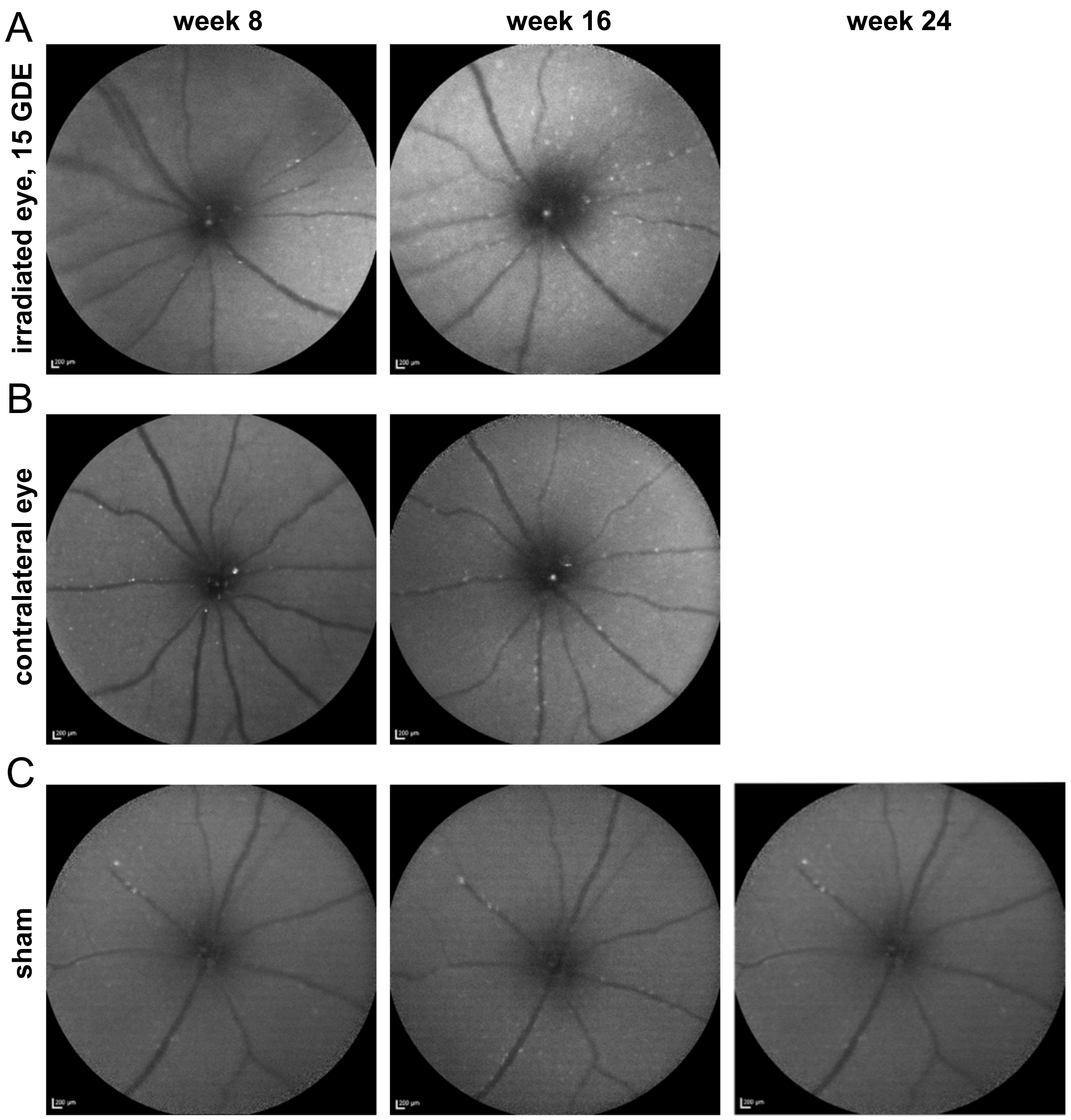

Supplementary Figure S1 Autofluorescence imaging of the retina of the wild-type genotype after proton irradiation, time-course of fundus autofluorescences after proton irradiation in the irradiated eye (A), in the contralateral eye (B) and after sham procedure (C).
