# Supplementary material for: Radiation Retinopathy: Microangiopathy-Inflammation-Neurodegeneration
Source: Cells. 2025 Feb 18;14(4):298. doi: 10.3390/cells14040298 (PMC11853965; doi:10.3390/cells14040298)
Supplement: Supplementary file 1 [file cells-14-00298-s001.zip › Figure S2.pdf]

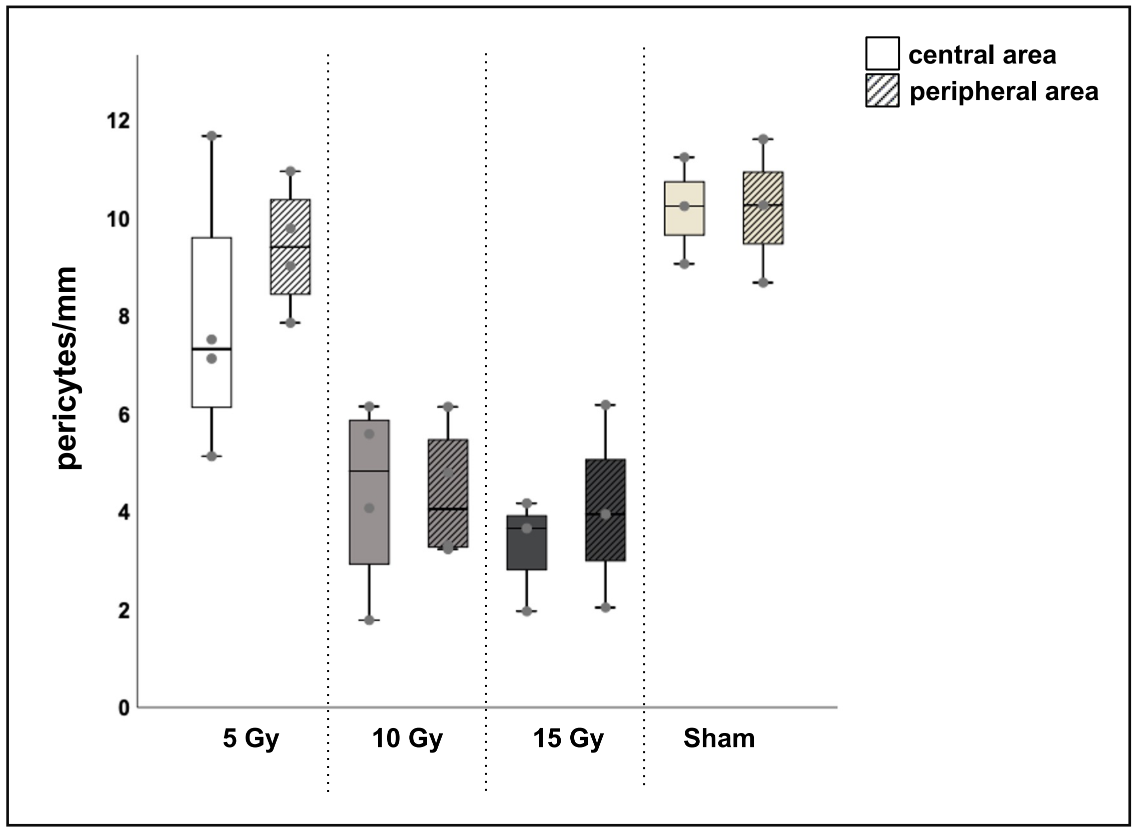


**Supplementary Figure S2** Number of pericytes per mm in the central versus peripheral area, no significant difference ( p > 0,05).
