# Supplementary material for: Radiation Retinopathy: Microangiopathy-Inflammation-Neurodegeneration
Source: Cells. 2025 Feb 18;14(4):298. doi: 10.3390/cells14040298 (PMC11853965; doi:10.3390/cells14040298)
Supplement: Supplementary file 1 [file cells-14-00298-s001.zip › Figure S3.pdf]

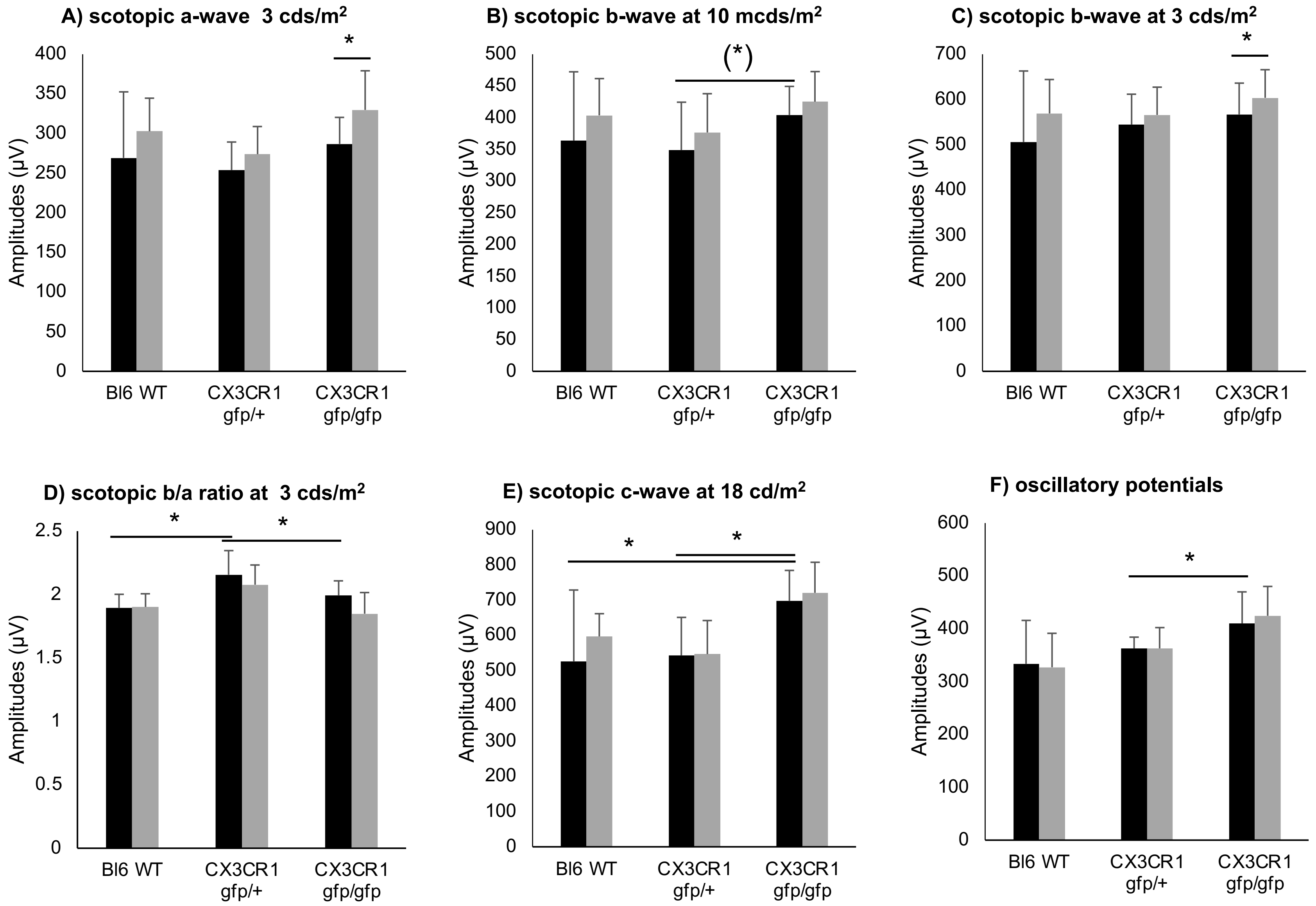

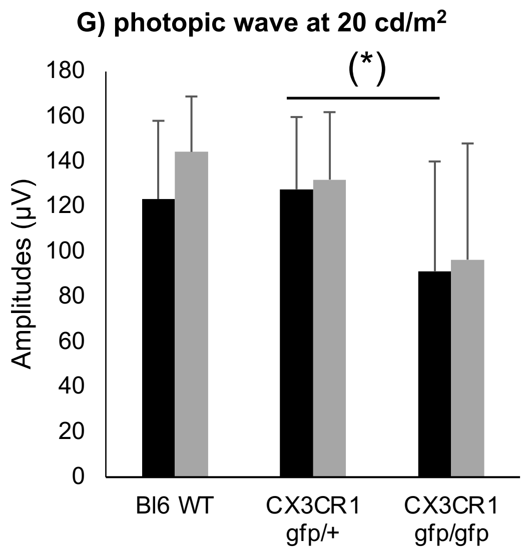

Supplementary Figure S3 Comparison of the Ganzfeld-ERG parameters of the irradiated eyes (black bar) in relation to the contralateral side (grey bar) for all genotypes 24 weeks after proton irradiation with 15 GDE:

A) significant difference between irradiated and non-irradiated eyes of homozygous Cx3cr1$^{gfp/gfp}$ mice (p = 0,007),

B) tendency difference between the irradiated and non-irradiated eyes of homozygous Cx3cr1$^{gfp/gfp}$ and heterozygous Cx3cr1$^{gfp/+}$ mice (p = 0,072),

C) significant difference between the irradiated and non-irradiated eyes of homozygous Cx3cr1$^{gfp/gfp}$ mice (p = 0,05),

D) significant difference between the irradiated eyes of heterozygous Cx3cr1$^{gfp/+}$ and wild-type (p = 0,003) or homozygous Cx3cr1$^{gfp/gfp}$ (p = 0,049) mice,

E) significant difference between the irradiated eyes of homozygous Cx3cr1$^{gfp/gfp}$ and heterozygous Cx3cr1$^{gfp/+}$ mice (p = 0,007) or wild-type mice (p = 0,043),

F) significant difference between the irradiated eyes of homozygous Cx3cr1$^{gfp/gfp}$ and heterozygous Cx3cr1$^{gfp/+}$ mice (p = 0,018),

G) tendency difference between the irradiated and non-irradiated eyes of homozygous Cx3cr1$^{gfp/gfp}$ and heterozygous Cx3cr1$^{gfp/+}$ mice (p = 0,058).
