# Supplementary material for: Radiation Retinopathy: Microangiopathy-Inflammation-Neurodegeneration
Source: Cells. 2025 Feb 18;14(4):298. doi: 10.3390/cells14040298 (PMC11853965; doi:10.3390/cells14040298)
Supplement: Supplementary file 1 [file cells-14-00298-s001.zip › Figure S4.pdf]

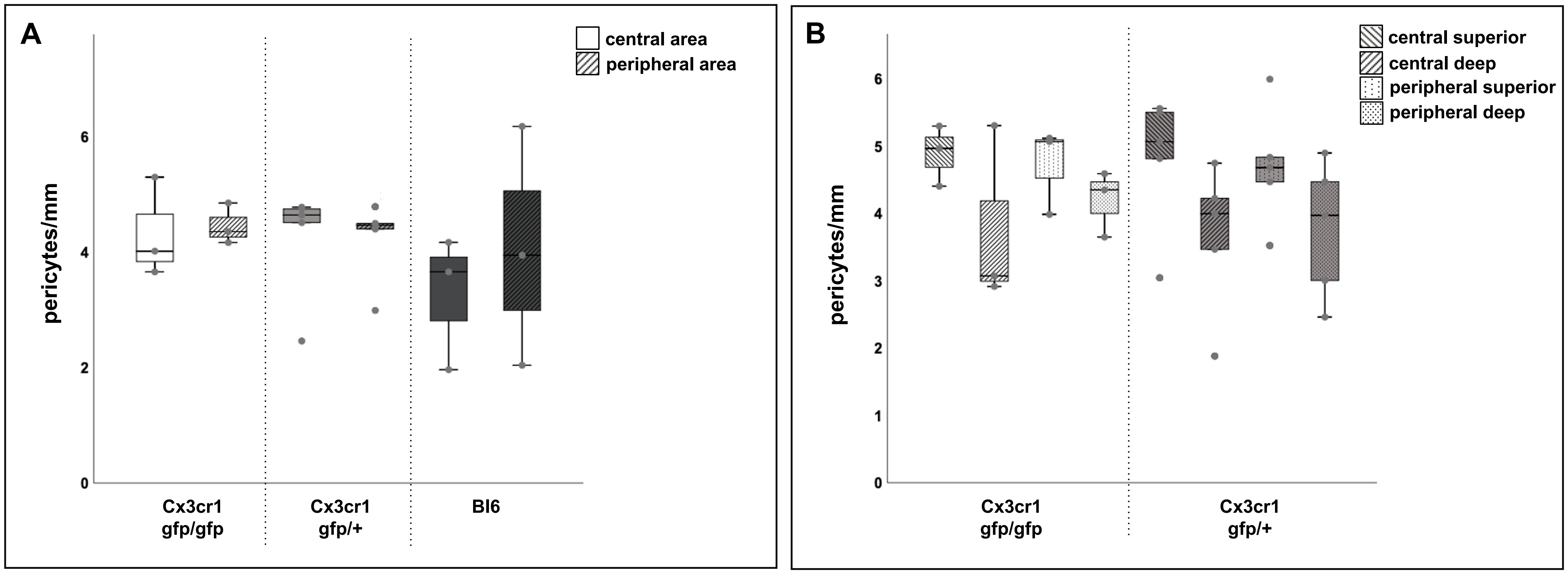

Supplementary Figure S4 Comparison of the number of NG2-positive cells between the areas, layers and genotypes in the irradiated eye,

A) number of pericytes per mm vessel length 24 weeks after proton irradiation, comparison of the areas, no significant difference between the number of pericytes per mm in the central and peripheral area, no significant difference between the genotypes,

B) number of pericytes per mm vessel length 24 weeks after proton irradiation, comparison of the superficial and deep vascular layers concerning the irradiated eye, no significant difference between the genotypes or layers.
