# Supplementary material for: Radiation Retinopathy: Microangiopathy-Inflammation-Neurodegeneration
Source: Cells. 2025 Feb 18;14(4):298. doi: 10.3390/cells14040298 (PMC11853965; doi:10.3390/cells14040298)
Supplement: Supplementary file 1 [file cells-14-00298-s001.zip › Figure S5.pdf]

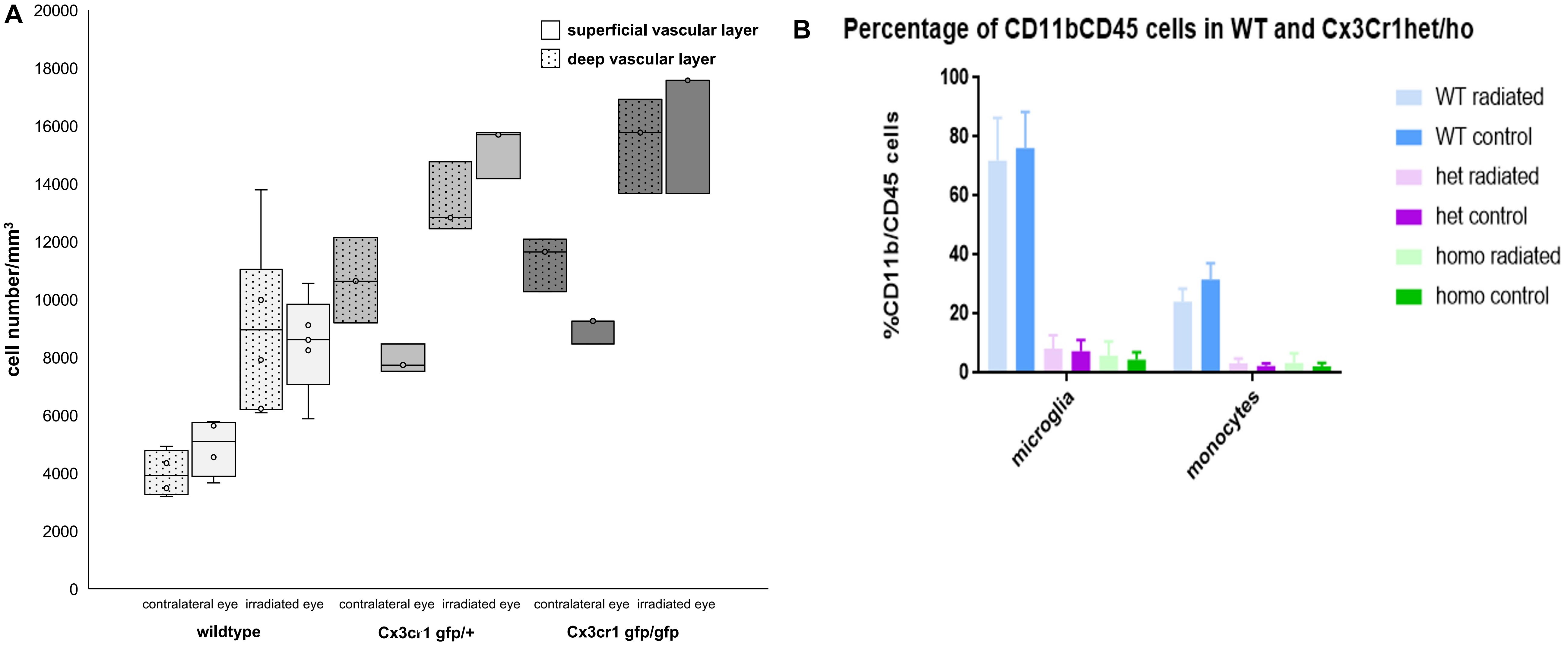


**Supplementary Figure S5**

A) Number of Iba1-positive cells per cubic millimeter, comparison of the vascular layers, no significant difference between the superficial and deep vascular layer in the irradiated eye or on the contralateral side,
tendency: on the irradiated side increased accumulation of Iba1- positive cells in the superficial vascular layer in the retina of the Cx3cr1gfp/gfp genotype than in the retina of the Cx3cr1gfp/+ genotype (p = 0,075).

B) FACS-Analysis, WT (n=5) Cx3cr1 heterozygote (n=7) and homozygote (n=10) females 6 months after irradiation: there is a significant decrease of CD11bCD45 positive cells in the het and ho compared to WT. There is no irradiation - effect seen on the cell populations.
